# Daily functioning and dementia

**DOI:** 10.1590/1980-57642020dn14-020001

**Published:** 2020

**Authors:** Gabriele Cipriani, Sabrina Danti, Lucia Picchi, Angelo Nuti, Mario Di Fiorino

**Affiliations:** 1 MD,Versilia Hospital, Neurology Unit, Lido di Camaiore (Lu), Italy.; 2 MD, Versilia Hospital, Psychiatry Unit, Lido di Camaiore (Lu), Italy.; 3 PhD, Clinical and Health Psychology Unit, Hospital of Pontedera, Pontedera (PI), Italy.; 4 PsyD, Clinical Psychology Unit, Hospital of Leghorn, Leghorn (LI), Italy.

**Keywords:** activities of daily living (ADLs), dementia, functional abilities, instrumental activities of daily living (IADLs), atividades da vida diária (AVD), demência, habilidades funcionais, atividades instrumentais da vida diária (AIVD)

## Abstract

Dementia is characterized by a decline in memory, language, problem-solving and in other cognitive domains that affect a person’s ability to perform everyday activities and social functioning. It is consistently agreed that cognitive impairment is an important risk factor for developing functional disabilities in patients with dementia. Functional status can be conceptualized as the ability to perform self-care, self- maintenance and physical activity. A person with dementia usually requires help with more complex tasks, such as managing bills and finances, or simply maintaining a household. Good functional performance is fundamental for elderly people to maintain independency and avoid institutionalization. The purpose of this review is to describe functional changes in demented patients, evaluating the variability in subgroups of dementias.

## Resumo.

Demência é caracterizada por declínio na memória, linguagem, resolução de problemas e de outros domínios cognitivos que afetam a capacidade de realização de atividades cotidianas e atividades sociais. É consensual que o comprometimento cognitivo é um importante fator de risco para o desenvolvimento de incapacidades funcionais em pacientes com demência. O status funcional pode ser conceituado como a capacidade de realizar autocuidado, automanutenção e atividade física. Uma pessoa com demência geralmente requer ajuda para tarefas mais complexas, como gerenciar contas e finanças, ou simplesmente realizar atividades domésticas. Um bom desempenho funcional é fundamental para que os idosos mantenham a independência e evitem a institucionalização. O objetivo desta revisão é delinear alterações funcionais em pacientes com demência, valorizando os subgrupos variados de demências.

Dementia constitutes a multifactorial process^1^ that is always associated with cognitive decline and impaired functioning. As the disease progresses, people living with dementia experience, in addition to impaired cognitive functions, gradual dysfunction and loss of individual autonomies. Besides decline in memory and/or other cognitive domains, the criteria for diagnosis of dementia require loss of functional reserve and pejoration in functional status.^2^ An important quality of life component from elderly people’s perspective is functional indepen dence. When older people show functional loss, they experience a variety of negative outcomes, such as higher rates of use of hospital services, institutionalization, and increased risk of death.^3^ The progression of healthy aging to dementia must be considered a continuum, both in terms of the slow manifestation of the impairment of cognitive functions, as well as functional limitation.^4^ Originally, mild cognitive impairment (MCI) was considered a condition in which someone has minor cognitive decline, not severe enough to interfere significantly with daily life and function. As a result, some authors proposed introducing minor functional disability among the criteria for diagnosing MCI.^5^ However, many years can separate the onset of disease, perceived only through mild impairments in everyday living, the evolution from the date of clinical diagnosis and the earliest functional deficits as patients advance to mild dementia, are often difficult to characterize.^6^ Furthermore, there are age and gender differences in functional limitations. Subjects with a low level of formal education can show early deficits in cognitive performances, as well as functional abilities.^7^ Data regarding differences in functional limitations by dementia subtype are limited and conflicting. The aim of this literature review is to describe functional disabilities in dementia and to understand the individual variability of limitations in the heterogeneity of the disease.

## METHODS

A literature review (Cochrane Library and PubMed databases) was carried out (only upper time limit: 2019) on daily functioning and dementia. We retrieved: 207 articles by using the search terms “activities of daily living” and “dementia”; 47 articles using the terms “instrumental activities of daily living” and “dementia”; 18 articles using the terms “daily functioning” and “dementia”; and 21 articles using the terms “functional abilities” and “dementia”. Publications found through this indexed search were reviewed and manually screened to identify relevant studies. We manually added relevant articles identified through other sources (i.e. Google Scholar and key journals). At the end of the process, 81 articles and chapters of books were included in our qualitative evidence synthesis.

## FUNCTIONAL ASSESSMENT: ACTIVITIES OF DAILY LIVING AND INSTRUMENTAL ACTIVITIES OF DAILY LIVING

Useful screening techniques or instruments can provide valuable clinical and psychosocial information and can expedite the efficient assessment of patients once they are routinely utilized. Therefore, the assessment of functional disability and its severity must be carried out through measurement scales both in the general population and in dementia.^8^^,^ 9 The instruments for assessing functional ability are divided into two levels, from the more basic (Activities of Daily Living - ADLs) to the more advanced activities necessary for self-care (Instrumental Activities of Daily Living IADLs). The activities that fall into the category of ADLs, involve all tasks that are needed to be performed in order for the patient to survive comfortably: mobility, toilet and bathing/continence, personal hygiene, dressing, and feeding; IADLs, on the other hand, are more complex than ADLs. They include transportation, shopping, preparing meals, managing households, managing finances, using communication devices, and managing medication. Performance of basic and instrumental activities of daily living depends upon the integrity of different cognitive (e.g., reasoning, planning), motor (e.g., balance, dexterity), and perceptual (including sensory) functions. Nevertheless, at present there is no “gold standard” available for ADL and IADL assessment in dementia, despite the fact that functional decline is an essential part of the diagnostic criteria for dementia.^10^^,^ 11 In clinical practice, information about everyday activities is typically ascertained by asking a patient or his/her caregiver to report everyday functioning. It has been suggested that family caregivers who are depressed and feel burdened may be inaccurate and underestimate the patient’s actual functional capacity.^12^ According to Loewenstein et al.,^12^ caregivers significantly overestimate the ability of impaired dementia patients to tell time, identify currency, make change for a purchase, and utilize eating utensils. The selection of a functional assessment instrument typically depends upon the severity of the dementia population being evaluated. Many assessment tools are available to help the clinician detect and monitor improvement in IADLs and ADLs in the elderly. The Katz ADL Index^13^ was first developed in an effort to find a way to assess function and how it changes over time in the elderly. It is an ordinal index designed to assess physical functioning using a dichotomous rating (dependent/independent) of six ADLs in hierarchical order of decreasing difficulty as follows: bathing, dressing, toileting, transferring, continence, and feeding, rated on a scale of independence. An appropriate instrument for assessing independent living skills was developed by Lawton and Brody in 1969 to assess the more complex ADLs necessary for living in the community: eight domains of function for women (ability to telephone, shopping, food preparation, housekeeping, laundry, mode of transportation, responsibility for own medication, ability to handle finances), but only five for men (food preparation, housekeeping, and laundry were excluded).^14^ The administration time of the Lawton IADL is 10-15 minutes and it is easy to administer. The patient or a knowledgeable family member or caregiver may provide answers. The higher the score, the greater the person’s abilities. A summary score ranges from 0 (low function, dependent) to 8 (high function, independent) for women, and 0 through 5 for men. As the items of the Lawton IADL scale conform to a formal hierarchy, the most “difficult” items such as “shopping” and “food preparation” can act as sensitive indicators of impending disability in the other activities.^15^ Fields et al.^16^ found that when using a caregiver-report measure, problems in bathing and grooming appeared first, whereas eating was the last to be affected. Elderly aged 80 or older are more than twice as likely to have limitations than those aged 65 to 74. The impairment of individual activities develops sequentially (i.e., housework, transportation, shopping, meal preparation, finances).^17^ IADL impairment is reported to develop earlier in dementia and has a higher prevalence, and stronger correlations with cognition than ADL,^18^ whereas basic ADL declines are often not present until later dementia stages.^19^ One study demonstrated that diminishing IADLs predicted a clinical diagnosis of dementia 10 years beforehand and suggested it as an early screening tool.^10^ Among all ADLs, bathing impairment may be associated with the highest risk of future institutionalization.^20^

## RELATIONSHIP BETWEEN FUNCTIONAL IMPAIRMENT AND COGNITIVE DECLINE

Cognition is a term referring to the mental processes involved in gaining knowledge and comprehension. These processes include thinking, knowing, remembering, judging and problem-solving. These cognitive domains are closely associated with the ability to perform everyday functions.^21^ Longitudinal studies of persons at risk for dementia have demonstrated the initial clinical presentation of the disease is one in which there are cognitive deficits measurable with performance-based tests, but with no evident deficit in activities of daily living. According to anecdotal reports, many of those with poor performances on mental status examinations may exhibit an essentially normal life. For this reason, it is essential to establish the degree of correlation between performance on a cognitive test and ability to function in daily life. While functional assessment typically addresses specific content areas, neuropsychological assessment normally focuses on patterns of relatively impaired and preserved generic, cognitive, perceptual, and motor abilities, such as language, memory, spatial ability, problem-solving ability, and perceptual-motor skills. The relationship between these two domains is complex, since a particular functional content area usually involves a number of the specific skills typically evaluated by neuropsychological tests. Neuropsychological assessment can be a reasonably good predictor of those activities of daily living that place relatively heavy demands on reasoning, memory, and related intellectual abilities. Executive function^22^ and memory have been shown to have specific relationships to functional limitations. Individuals with marked executive dysfunction are likely to have significant difficulty carrying out such complex tasks as managing a complicated medication regimen, preparing a meal involving multiple ingredients and steps, or balancing a check-book.^23^ Barberger-Gateau et al.^9^ performed a study identifying which IADL items are more specifically related to cognitive impairment as assessed by the MMSE, in a representative sample of French elderly community dwellers. The authors affirmed that, when age, sex, educational level, and all Lawton’s scale items are simultaneously taken into account, only items A (telephone), G (medication) and H (budget), plus F (transportation) for women, are significant. More recently, Brown et al.^24^ investigated whether the MMSE was associated with functional performance as measured by the Functional Independence Measure (FIM)^25^ (the FIM is an 18-item, clinician-reported scale that assesses function in six areas including self-care, continence, mobility, transfers, communication, and cognition). The MMSE scores derived for inpatients with suspected dementia were significantly associated with the inpatients’ total FIM and cognition subscale scores. The Short Portable Mental Status Questionnaire (SPMSQ),^26^ a mental status test that emphasizes memory, orientation, and calculation, was deemed an inadequate predictor of self-care capacity in nursing home patients.^27^ A strong association was found between the MMSE and the Disability Assessment for Dementia (DAD),^28^ in particular DAD total score.^29^ There was an association between functional status and visuospatial performances among AD samples.^30^ A significant and specific relationship was found between measures of visual object form discrimination and the adequacy of performance of IADLs that require visual processing; no similar relations emerged between other visual perceptual abilities and IADLs.^31^ Damage to the frontal lobe, the prefrontal cortex in particular, was associated with problems in successful completion of goal-directed behaviour and with a variety of neuropsychiatric syndromes: Instrumental Activities of Daily Living (IADLs) such as using transportation, managing financial matters, and organizing a household require the planning strategy and adjustment capacities of the dorsolateral prefrontal cortex.^32^ Executive dysfunction serves as a predictor of IADL impairment both in dementia and, in general, in geriatric patients.^33^ Abilities that fall under executive function include goal planning, initiating and executing actions, multitasking, switching between tasks, monitoring, and inhibiting habitual behaviours when presented with unexpected events.^34^ Mariani et al. affirmed that performance of tasks such as shopping and medication management, is mainly related to more severe cognitive dysfunction and lower executive abilities, supporting the connection between executive function, global cognition, and IADLs.^35^ Mayo et al.^36^ hypothesized that there is a relationship between judgment/problem solving and functional status: findings showed that cognition moderated a strong relationship between functional status and judgment/problem solving among individuals with dementia, with lower reported functional performance predicting poorer judgment/problem solving.

## RELATIONSHIPS BETWEEN FUNCTIONAL DECLINE AND BEHAVIOURAL DISTURBANCE

Performance on cognitive testing predicts only a modest proportion of the variance in functional abilities in persons with cognitive impairment, indicating that other variables also predict function. Behavioural disturbances, such as apathy,^37^ depression,^38^ and delusions,^39^ are frequent bothersome characteristics of people living with dementia that increase with disease severity. Additional problem behaviours among late-stage dementia patients include wandering,^40^ disruptive vocalizations,^41^ and inappropriate sexual behaviours.^42^ An understanding of which neuropsychiatric symptoms are most strongly associated with functional disability may encourage health care providers and loved ones to vigilantly monitor for their presence and aggressively treat these symptoms to reduce their potentially modifiable effects on function. One study strongly indicated the close relationship between behavioural and functional impairment, especially for IADLs that deteriorate in the early stages of the disease and are hierarchically more complex than ADLs.^43^ Given the consistent associations between behavioural disturbances and functional disability, researchers have attempted to determine their relative strengths in predicting everyday functioning in persons with cognitive impairment.^44^ The development and severity of behavioural disorders are not always correlated with poor cognitive symptoms and functional disability; changes in behaviour may be determined by other factors, such as the variety of clinical signs and symptoms of dementia, personality traits of patients, the social support required and available, and the capacity to manage stress of caregiver.^45^ Associations have been reported between behavioural disturbance and ADLs, such as toileting and hygiene,^46^ and IADLs, such as managing medications and finances.^44^ Different types of neuro-behavioural changes have been associated with functional disability.^47^ Some features of apathy and depression overlap. In persons with cognitive impairment, apathy may be more common than depression, which is characterized by guilt, sad mood, hopelessness and poor self-concept. Apathy is characterized by loss of interest, social withdrawal, and generally decreased motivation, initiation, and persistence in the absence of low mood or depressive thought patterns. Apathy has been associated with impairments in planning, initiating and executing IADLs,^43^ while depression was only associated with impaired initiation and planning. Depression has been shown to be a strong predictor of functional difficulty.^48^ Depression reduction was associated with benefits to non-mood outcomes of importance to patients who had dementia and their caregivers.^49^ The most striking benefit involved ADLs, with stabilization, and perhaps some reversal of ADL decline. Anxiety and aberrant motor disturbance may also be an important risk factor for functional disability.^50^

## ALZHEIMER’S DISEASE

Alzheimer’s disease (AD) is an age-related progressive neurodegenerative disorder representing the most common form of dementia, that ultimately leads to death due to complications of the disease or to age-related mortality. Its prevalence increases exponentially between the age of 65 and 85 years.^51^ The diagnosis of AD requires that patients display both cognitive and functional deterioration. There is robust evidence and consensus that the onset of the symptoms of AD is so insidious that neither family nor patient can pinpoint the exact date of onset. It is now clear that there is continuum from psychosomatic individuals, to those with MCI, to patients with dementia, and that the pathological hallmarks of the disease are present years before cognitive symptoms are recognized.^52^^,^ 53 Functional impairment is a core symptom of AD, significantly impacting the quality of life of persons with AD as well as of their family members and caregivers.^29^ Data support the concept that decline in cognition is later reflected in functional deficits.^54^ Researchers have studied the temporal relationship of cognitive deficit and functional impairment in AD. The correlation observed supports the hypothesis that, as disease progresses, cognition becomes more clearly related to function and the two measures become more strongly associated. IADLs, specifically finance and medications or outings, are the first to decline with memory deterioration and evolving of behavioural changes; decline in ADLs follows when, gradually, dementia progresses and becomes severe and executive dysfunction becomes evident.^55^

Furthermore, IADLs requiring higher neuropsychological functioning seem to be more severely affected than ADLs.^56^ Observations suggest that the influence of poor cognitive function on impairments in everyday activities becomes more significant as the disease progresses from MCI to the early symptoms of dementia, and then to the severe form of AD, with the magnitude of correlations depending on the complexity of the functional task.^57^^,^ 58

## FRONTOTEMPORAL DEMENTIA

Frontotemporal dementia (FTD) was first described by Arnold Pick (1851-1924), a Czech psychiatrist, neurologist and neuropathologist, in 1892. It covers a wide range of different conditions, representing a group of neurodegenerative dementias affecting the frontal and/or temporal lobes relatively selectively, even in later stages of the disease. It is most often diagnosed between the ages of 45 and 65. Therefore, FTD has a substantially greater impact on work, family, and economic burden faced by families than AD. There are three subtypes of FTD: the behavioural-variant FTD (bvFTD), the most common presentation of the three variants, characterized by behaviour changes, emotional blunting, loss of empathy, and personality decline, progressive non-fluent aphasia (PNFA) characterized by agrammatism, effortful speech, alexia, and agraphia, semantic dementia (SD) with loss of semantic knowledge and inability to match certain words with their images or meanings.^59^ FTD impinges markedly on everyday function, but studies evaluating functional status in the course of FTD are sparse. Functional difficulties depend on the clinical subtype of dementia and its severity. These differences are recognizable even after controlling for age, education, and disease duration.^60^ The bvFTD patients proved the most impaired group and this deficit was particularly evident in ADLs. The PNFA group, despite being the least impaired overall, had subtle, but definite, problems beyond language-based IADLs. Similarly, the SD group was not only affected in language-based activities, but impairment extended to IADLs, such as “leisure and house chores,” “going on an outing,” and “meal preparation.” It should be highlighted that bvFTD may sometimes have a catastrophic effect on ADLs, which may not be reflected in cognitive test scores.^61^ Patients with SD remain relatively independent in everyday tasks for a much longer period of time, in line with a much more protracted disease progression.^62^ With worsening of the disease, functional outcomes become similar in all FTD variants.^63^ Stereotypical behaviour and ADL decline are associated with disability in patients with bvFTD.^63^ However, little is known about the rate of deterioration of functional activities in FTD patients over a 12-month period and if the decline is associated with changes on general tests of cognitive function.^62^ The rate of deterioration for bvFTD and PNFA patients are more marked than those reported in studies of AD patients, whereas SD patients decline at a similar rate to AD.^64^^,^ 65 Some authors have investigated the relationship between emotion and social skills assessed by ADL scales.^66^ In fact, decision-making may be affected by the inability to use emotional cues to bias behaviour in social situations. The researchers demonstrated that the ability to perform ADLs is independent of impaired emotion in FTD.

## DEMENTIA WITH LEWY BODIES

Dementia with Lewy bodies (DLB) is a progressive, degenerative dementia of unknown aetiology and complex clinical picture. Fluctuating cognitive function is a relatively specific feature of a person with DLB; other clinical features are recurrent visual hallucinations, behavioural problems, extrapyramidal symptoms (typically including rigidity, bradykinesia, and gait instability), and changes in autonomic body functions, such as blood pressure control, temperature regulation, and bladder and bowel function.^67^ It is characterized by cellular inclusions called Lewy bodies in the cytoplasm of cortical neurons, the limbic system and brainstem structures. Lewy bodies are abnormal, eosinophilic spherical structures, resulting in neuronal cytoplasmic inclusions composed of aggregates of alpha-synuclein, a synaptic protein. Little is known about how much individual cognitive, behavioural and motor problems influence functional performance in patients suffering from DLB. In the elderly with motor disorders, functional impairment is particularly significant, especially for continence and walking^68^ (up to 75% of DLB patients are reported to have extrapyramidal motor symptoms during the illness). Differences in functional changes between people with DLB and AD tend to manifest in the early stages of the diseases, whereas these difference tend to disappear in advanced stages. According to Stavitsky et al.,^69^ at first evaluation, patients with DLB were significantly more impaired on measures of ADL and showed greater dependence on caregivers, but functional changes over time were similar in the two groups. Similarly, another study found no differences in the frequency of development of severe functional impairment between patients with AD and those with DLB.^70^ In DLB, motor disability best predicts IADL ratings, with severity of cognitive impairment adding predictive value.^71^ An explanation is that motor disability in DLB serves as a proxy for the general integrity of the basal ganglia, and cognitive impairment associated with basal ganglia dysfunction (e.g., executive dysfunction) contributes to the IADL deficits in DLB.

## PARKINSON’S DISEASE DEMENTIA

Parkinson’s disease (PD) is a long-term, progressive, degenerative nervous system disorder that affects a wide range of functions. It is generally accepted that the main pathological feature of this condition is damaged dopaminergic nigrostriatal pathways with decreased concentration of dopamine in the compact zone of the substantia nigra. Resting tremor is the most common clinical feature. Bradykinesia, rigidity and postural instability are often detectable. It is now recognized that PD is much more than a motor disorder. In the course of the illness, autonomic symptoms, anxiety, mood disorder and cognitive changes are often observed.^71^ PD patients may have deficits in multiple cognitive areas from the initial stages of the disease progressing ranging from subtle symptoms (mild cognitive impairment in PD - PD-MCI) to clear cognitive alterations (PD dementia - PDD). PDD has been increasingly better recognized, probably because persons with PD survive for longer than before owing to modern treatment. Different cognitive profiles may exist within PD, but cognitive deficits associated with PD mainly involve executive functions, memory, attention and visuo-spatial functions, but other cognitive functions may also be impaired, often as a secondary consequence of the primary executive disorder.^72^ The aetiology of dementia in PD has not yet been fully established. The rate of early disability in PD patients is associated with declining cognitive function.^73^ We know that dysfunction in everyday activities occurs early in the course of the disease and its detection is important for patients to fully understand their substantial difficulties in daily life. For example, people with PD show compromise in housekeeping, managing money, and preparing meals.^74^ While it has been well documented that the extrapyramidal syndrome is associated with impairment in basic ADLs,^75^^,^ 76 Rasovska and Rektorova also observed highly significant correlations between IADL and functional disability.^77^ Axial non-dopaminergic symptoms (such as postural instability and gait difficulty) influence IADLs; this is more evident in PDD patients than in persons suffering from PD without dementia.^77^ However, a recent study demonstrated that cognitive deficits contribute to a greater functional decline in ADL performance.^78^ More specific to PD than AD is impairment of executive functions as the hallmark feature of cognitive dysfunction.^79^ Patients with PD showing executive impairment scored lower on instrumental self-maintenance, the use of new devices, and life management compared to those not presenting executive function impairments.^73^ Financial capacity represents a cognitive set of knowledge and skills that has a special characteristic as an IADL. It correlates poorly with motor function and is usually clearly compromised in persons with PDD.^80^ Disability in IADLs is particularly correlated with PD duration.^79^

## PROGRESSIVE SUPRANUCLEAR PALSY

Steele, Richardson and Olszewski first described progressive supranuclear palsy (PSP). It is an uncommon degenerative neurological disorder representing the most common form of atypical parkinsonian syndrome. It is a debilitating disease. Symptoms usually emerge at50-60 years of age, with onset ranging from the early forties to late eighties. PSP symptoms include progressive, early-onset postural instability, frequent (unexplained) falls, impaired eye movement (vertical supranuclear gaze palsy), axial (involving neck or trunk) rigidity and speech/swallowing difficulties. Clinically, the presence or absence of functional impairment may dictate a diagnosis of dementia or MCI, respectively.^81^ Affected individuals frequently experience personality changes and memory and executive attention deficits.^82^ Mood and behavioural changes may occur. People with PSP may become irritable, depressed or apathetic; they may also become more impulsive in their decision-making.^83^ Functional disability is high in patients with early-stage PSP.^84^ Duff et al.^84^ examined functional profiles of patients with early-stage PSP: 100% of the participants in their study underperformed on all scales, suggesting at least some functional disability.

## HUNTINGTON’S DISEASE

Huntington’s disease (HD), sometimes called Huntington’s chorea, is an incurable, lethal, genetically inherited neurodegenerative disorder caused by an expansion of a repeating cytosine-adenine-guanine (CAG) triplet series in the huntingtin gene on the short arm of chromosome 4, resulting in impairment of multiple domains. The illness is characterized by motor, cognitive, and psychiatric symptoms, which begin insidiously and progress over many years, until the death of the individual. It is thought that the variability in disease severity and rate of progression among people with HD is linked to the genetic mutation causing the disease. Patients and their families note the progression of the disease in different ways, as symptoms are present at different times from person to person; however, it is difficult to divide physical and mental traits.^85^ The cognitive deficits can precede the appearance of motor symptoms by as much as 20 years, although they most often emerge in the 10 years leading up to clinical diagnosis of the disease. Because the disease affects the frontal lobes of the brain, planning ability, judgment and decision-making are affected; memory appears especially affected, with problems occurring for both verbal and non-verbal memory; motor functions are disrupted, which interferes with speech and coordination.^85^ Individuals with mildly deteriorating conditions may be able to carry on their ‘normal’ life for many years and continue to function well in their job and with hobbies and activities, but at some point they will become disabled and need help to carry out activities of daily living. The most common functional declines show relationships with behavioural changes, motor functioning, and cognitive deficits. In particular, the latter deficits are responsible for functional losses in managing finances, working performance and driving.^86^ Certain changes in cognitive abilities are characteristic of HD and can significantly affect the lives of individuals with the disease. For example, cognitive changes may affect the ability of a person with HD to work, manage a household or properly care for him or herself regardless of motor impairment.^87^ Furthermore, in the early stages of HD (duration 0 to 5 years), cognitive deterioration is considered an important factor that determines the loss of functional ability.^88^ The role of emotional changes needs further elucidation. Regarding neuropsychological performance and depressive symptomatology, these are considered predictive factors of functional disability.^86^ Behavioural problems associated with the disease are thought to contribute significantly to dysautonomia in daily functions: the significant loss of motivation, absence of initiative, and irascibility present in some Huntington’s chorea individuals may affect their ability to perform basic activities of daily living, even if cognitive and motor functioning remain intact.^87^

## VASCULAR DEMENTIA

Vascular dementia (VaD) is an umbrella term for a group of conditions that recognize vascular brain damage of ischemic, haemorrhagic, or hypoxic type as a common pathophysiological event. Its prevalence increases exponentially with age and its risk doubles every 5.3 years. Clinical^37^ manifestation may be cortical or subcortical. Cortical manifestations include cognitive and behavioural symptoms, with or without sensory or motor deficits. Subcortical VaD patients have sensory and motor deficits, gait disorders, dysphagia, dysarthria, extrapyramidal signs, urinary incontinence, emotional lability, impairment of attention and executive function with slowing of information processing. Frequently, small infarcts remain clinically “silent”, producing no apparent symptoms, while larger infarcts are more likely to produce impairment. This is especially true for subcortical white matter ischemic events, which may not produce cognitive dysfunction until a particular threshold has been exceeded.^89^ There is limited information on functional limitations in VaD. This fact may, in part, be due to the clinical heterogeneity in subgroups of patients.^34^ Functional status among people with VaD is often conditioned by sensory and motor deficits, but other underlying factors such as perceptual and mood changes, apathy, and even urinary incontinence, have a negative impact on both ADLs and IADLs. In addition, the co-presence of other diseases such as diabetes, peripheral arteriopathies, and heart failure further reduces functional capacity in VaD patients.^55^ Therefore, it is not easy to ascertain the main origin of disability in everyday functioning. Individuals with VaD have significant limitations in ADLs (eating, toileting, and transferring) and in IADLs (grocery shopping and cooking).^55^ IADLs are typically affected at earlier stages with worsening of memory and the development of behavioural disturbances, followed by a progressive decline in ADLs as executive function becomes more affected at later stages of the dementia.^10^ People suffering from VaD present functional impairment that differs from that presented by AD patients. However, in the scientific literature, there are data that contradict this assertion; in fact, some authors claim that the rate of progression of ADL deficits in VaD is slower than in AD, but that the impairment is qualitatively similar.^34^ Researchers have observed worse performance in almost all variables of both ADLs and IADLs in patients with subcortical ischemic vascular disease compared to those with AD: the fully adjusted model indicated that patients with subcortical ischemic vascular disease had worse performance in toilet use among ADLs and in laundry and ability to handle finances among IADLs than patients with AD.^90^ Associations between specific cognitive domains and functional disability have been studied and show that executive functions consistently predict everyday functioning in cognitively impaired older adults with VaD. This is especially true for IADL dysfunction in patients with VaD due to small vessel disease.^91^^,^ 92 Given the consistency of reports indicating frequent and prominent executive dysfunction among patients with VaD allied to the increasing evidence of its functional significance, evaluations of executive abilities are recommended for VaD patients, particularly those with VaD due to small vessel disease.^34^ Jefferson, et al.^93^ highlight the impact of executive processes on functional performance, such as using means of transport or managing money. Tasks such as cooking, housekeeping and managing finances are the most vulnerable to cognitive decline. Disturbances in ADLs may be considered unidimensional, but their clinical repercussion might vary according to the hierarchy or importance of the function affected. Changes in executive functioning and memory over a one-year period were predictive of IADL and ADL changes.^93^ Functional impairment is more severely impaired among those subjects with both cortical infarcts and white matter ischemia, but the differences were largely quantitative rather than qualitative. This finding was not observed for both basic and instrumental activities, as significant group differences were evident only on the more complex instrumental behaviours such as managing money and shopping.^89^

## CONCLUSIONS

Dementia goes beyond cognitive impairment, also encompassing functional disability. With disease worsening, physical, cognitive and clinical problems accumulate and the pattern of loss follows a distinct progression. The first areas requiring external support in functional status are the IADLs and, over time, there is a need for support in performing ADLs. Expected functional decline may be an even more important issue for families than cognitive decline. Cognitive impairment is a condition with a high impact on the aetiology of disability, independently of other clinical variables, while impairment in functions of daily living worsens with clinical stage of dementia. However, data indicate that disability is significantly affected by comorbidity. Behavioural symptoms also play a role in deteriorating function. Executive function is a high complexity cognitive domain which comprises several functions required for the efficient execution of a cognitive process, enabling active retrieval of the information stored in long-term memory. A correlation between dysexecutive syndrome and poor functional status has been observed. Furthermore, it is clear that executive functioning measures are able to predict functional outcome. Knowing the stages of functional decline in dementia can help clinicians to make decisions regarding patients, considering that dementia affects each patient differently. It is important to make the necessary lifestyle adaptations, while remaining flexible about meeting needs as they evolve. Clinicians should be able to assess functional performance, where this information is integral to understanding health and for the optimal provision of clinical care and implementation of individual measures of rehabilitation designed to improve executive function.^94^
